# Inhibition of USP7 upregulates USP22 and activates its downstream cancer-related signaling pathways in human cancer cells

**DOI:** 10.1186/s12964-023-01320-z

**Published:** 2023-11-09

**Authors:** Keqiang Zhang, Ting Sun, Wendong Li, Yuming Guo, Aimin Li, Marcus Hsieh, Jinghan Wang, Jun Wu, Leonidas Arvanitis, Dan J. Raz

**Affiliations:** 1 Division of Thoracic Surgery, City of Hope National Medical Center, Duarte, CA USA; 2 Faculty of Health Science, University of Macau, Macau, China; 3 Division of Comparative Medicine, City of Hope National Medical Center, Duarte, CA USA; 4 Pathology Core of Shared Resources, City of Hope National Medical Center, Duarte, CA USA; 5 Department of Hepatobiliary and Pancreatic Surgery, East Hospital, School of Medicine, Tongji University, Shanghai, China; 6 Department of Pathology, City of Hope National Medical Center, Duarte, CA USA

**Keywords:** Deubiquitinase, USP7, USP22, SP1, c-Myc, p53, Targeted anticancer therapy

## Abstract

Deubiquitinases (DUBs) play important roles in various human cancers and targeting DUBs is considered as a novel anticancer therapeutic strategy. Overexpression of ubiquitin specific protease 7 and 22 (USP7 and USP22) are associated with malignancy, therapy resistance, and poor prognosis in many cancers. Although both DUBs are involved in the regulation of similar genes and signaling pathways, such as histone H2B monoubiquitination (H2Bub1), c-Myc, FOXP3, and p53, the interdependence of USP22 and USP7 expression has never been described. In the study, we found that targeting USP7 via either siRNA-mediated knockdown or pharmaceutical inhibitors dramatically upregulates USP22 in cancer cells. Mechanistically, the elevated USP22 occurs through a transcriptional pathway, possibly due to desuppression of the transcriptional activity of SP1 via promoting its degradation upon USP7 inhibition. Importantly, increased USP22 expression leads to significant activation of downstream signal pathways including H2Bub1 and c-Myc, which may potentially enhance cancer malignancy and counteract the anticancer efficacy of USP7 inhibition. Importantly, targeting USP7 further suppresses the in vitro proliferation of USP22-knockout (USP22-Ko) A549 and H1299 lung cancer cells and induces a stronger activation of p53 tumor suppressor signaling pathway. In addition, USP22-Ko cancer cells are more sensitive to a combination of cisplatin and USP7 inhibitor. USP7 inhibitor treatment further suppresses in vivo angiogenesis and tumor growth and induced more apoptosis in USP22-Ko cancer xenografts. Taken together, our findings demonstrate that USP7 inhibition can dramatically upregulate USP22 in cancer cells; and targeting USP7 and USP22 may represent a more effective approach for targeted cancer therapy, which warrants further study.

Video Abstract

**Supplementary Information:**

The online version contains supplementary material available at 10.1186/s12964-023-01320-z.

## Introduction

The ubiquitin–proteasome system (UPS) is involved in multiple fundamental cellular processes through regulating more than 80% cellular proteins’ degradation and function; and dysregulation of UPS has been implicated in the pathogenesis of many human diseases such as neurodegenerative disorders, viral diseases and cancer [1, 2]. The process of ubiquitination is highly dynamic and is reversed by the deubiquitinases (DUBs). As the main members of the DUBs family, ubiquitin-specific peptidases (USPs) are involved in numerous cellular processes including those frequently altered in carcinogenesis such as cell growth and survival; and increasing studies have indicated that dysregulated USPs represent potential targets for cancer treatment, and functional studies have also generally found that multiple DUBs are involved in these same processes, suggesting considerable redundancy in DUB-regulated processes [3]. Among these USPs, USP7 is one of the most well-characterized as a highly promising target for a variety of cancers, while increasing attention has been drawn to USP22 as another potential target for the development of anticancer therapeutics because of its multiple roles in cancer progress and immune response.

USP7, also known as herpes virus associated protease (HAUSP), regulates the deubiquitination of histone H2B monoubiquitinion (H2Bub1) [4] and the stability of multiple proteins including p53-MDM2 [5, 6], β-catenin [7] PTEN [8], and FOXP3/4 [9], and is involved in diverse cellular processes including DNA transcription, DNA damage response (DDR), epigenetic control of gene expression, immune response, and viral infection [10]. Notably, USP7 has been extensively studied for its ability to regulate the stability of p53 tumor suppressor and downstream signaling pathways that control cell cycle and apoptosis [5]. USP7 is frequently overexpressed in various human cancers including breast carcinomas, lung squamous cell carcinoma, lung large cell carcinoma, ovarian cancer, and chronic lymphocytic leukemia, and overexpression of USP7 protein is associated with high tumor grade, clinical aggressiveness, invasion, metastasis, and poor prognosis in cancer patients [6, 8, 10, 11]. As a result, USP7 has emerged as a promising target for cancer therapy. In the past two decades, dozens of potent and highly specific small-molecule inhibitors of USP7 have been developed, and there are several USP7 inhibitors in preclinical development [12, 13]. Studies in mice have demonstrated that pharmacological inhibition of USP7 suppressed the growth and metastasis, promoted cancer cell death in vitro and in vivo [14, 15]. Moreover, USP7 also plays an important role in regulating host immune response against cancer, and a recent study reported that P5091, a USP7 selective inhibitor effectively inhibited the growth of colon carcinoma xenografts through enhancing anticancer immunity via decrease of FOXP3-positive T regulatory cells (Treg) in tumor bearing mice [16], indicating USP7 inhibition may also potentially boost the host antitumor immunity.

USP22, a subunit of the distinct subcomplex called the deubiquitinating module in human SAGA (Spt-Ada-Gcn5-Acetyltransferase) complex, regulates both transcription initiation and enlogation controls by catalyzing removal of H2Bub1 [17, 18]. USP22 was initially identified as one component of a “Death-By-Cancer**”** 11-gene mRNA signature associated with poor prognosis in a variety of cancers [19]. USP22 is frequently overexpressed in breast, colon, lung, and other cancers [17, 20, 21], and overexpressed USP22 is associated with chemotherapy resistance in hepatocellular, breast, and colorectal cancers [22, 23]. USP22 is considered a putative cancer stem cell (CSC) marker which is critical for CSCs self-renewal in varous cancers [19, 24, 25], and a proangiogenic factor in the placental labyrinth [26]. USP22 is frequently overexpressed in lung cancer and associated with poor prognosis of lung cancer patients. In addition, USP22 knockout drastically suppresses CSCs maintenance, in vivo angiogenesis, growth, and metastasis of lung cancer, and significantly sensitizes lung cancer cells including CSCs to cisplatin and irradiation [27–29]. Additionally, USP22 affects DNA damage response (DDR), and USP22 is required for γH2AX deposition in response to DNA damage from irradiation [30]. Recent studies also demonstrate that USP22 plays a critical role in cancer immunosuppression through stabilizing FOXP3 activity in Treg [31] and programmed death-ligand 1 (PD-L1) in cancer [32, 33]. Therefore, like USP7, accumulating evidence indicates that USP22 also represents a promising target for cancer therapy, and USP22 knockdown markedly decreases cancer growth, induces apoptosis, and sensitizes cancer cells to chemo-radio and immune therapies [17, 34–36]. So far, USP22 inhibitors haven’t been reported in the literature.

Co-overexpression of USP7 and USP22 have been found in multiple cancers. Both USPs are associated with poor prognosis of cancer patients, and they are also involved in the regulation of several overlapping targets and signaling pathways such as p53/Foxp3/PD-L1/HIF1A and H2Bub1 which are critical to cancer development and therapy response. However, the interdependence between these two important USPs has never been reported. In this study, through screening known USPs inhibitors against USP22 DUB complex, we, for the first time, surprisedly found that pharmaceutical inhibition or knockdown of USP7 can dramatically upregulate USP22 protein in cancer cells independent of these cells’ p53 status. Moreover, we have further explored the therapeutic implications of this feedback loop and its underlying mechanisms in cancer cells. The findings of our study have strongly suggested that targeting both USP7 and USP22 may represent a novel, more effective therapeutic approach for cancer treatment, which warrants further study.

## Materials and methods

### Cell proliferation and compound treatment

Human lung cancer cell lines: A549 (p53 wild-type, K-Ras mutated), H1299 (p53-null, K-Ras mutated), and colon cancer cell line HCT116 (p53 wild-type, K-Ras mutated) were cultured in DMEM, RPMI, McCoy’s5A medium respectively supplemented with 10% FBS and 100U/ml penicillin and streptomycin. USP22 knockout (USP22-/-; USP22-Ko) lung cancer cells were generated from A549 and H1299 cell lines by CRISPR/Cas 9 system as previously described [27]. All cells were authenticated by Integrative Genomics Core by using short-tandem repeat polymorphism analysis before use. For cell proliferation and cytotoxicity of compounds, cells were seeded in 96-well plates in 4–6 replicates at densities of 2.0 × 10^3^ cells per well, after 24 h, compounds were added to wells respectively and further incubated with cells for 72 h. Then after, 10 µl of CCK-8 solution was added to each well and further developed for 2 h cell counting kit-8 as described previously [37]. The cell viability was calculated by the optical density (OD) values of treated groups/OD values of control groups (Vehicle/PBS) × 100%.

### Transfection, luciferase reporter assay and siRNA screening

USP22 promoter-driven firefly luciferase reporter plasmids (region from − 5085 to + 55) reported previously [38] or c-Myc (Myc/Max) response element renilla luciferase reporter (Addgene 118,069 [39]) and Renilla-luciferase/Firefly luciferase expressing plasmid with a ratio of 10:1 by using lipofectamine 3000. About 20,000 cells for each well were mixed with transfection master mix, and then seeded in 24 well plates. At 12-16 h post-transfection, cells were further treated with compounds for additional 48 h, and then cells were washed with PBS and lysed for 15 min in room temperature, lysis was analyzed by TECAN plate reader for both Firefly and Renilla luciferase activity according to the manufacturer’s instruction of Dual-luciferase reporter system (Cat #: E1910, Promega). Human USP7 specific siRNA (sc-41,521) was purchased from Santa Cruz Biology. pQFlag-USP7 WT puroR (Addgene plasmids #46,751) and pQFlag-USP7 CS puroR (and #46,752) encoding human USP7 deubiquitinase and catalytically inactive form of USP7 bearing C223S mutation were gifts from Gordon Peters [40]. RSV-Sp1 plasmid encoding human SP1 transcriptional factor was a gift from Robert Tjian (Addgene plasmid # 12,098). USP7 small-molecule inhibitors: FT671, HBX 41,108, and P5091 were purchased from MedChemExpress LLC (Monmouth Junction, NJ).

### Western blot and immunohistochemistry (IHC) analysis

Primary antibodies against β-actin, total and phosphorylated beta-Catenin, CD31, cleaved caspase 3, H2Bub1, MDM2, c-Myc, USP7, USP22, SP1, p53, and acetyl-p53 (Lys382, #2525) etc. were purchased from Cell Signaling Technology, Abcam, Santa Cruz Biotechnology or GeneTex respectively. Western blot was performed as described previously [27].

Both Multiplex and single IHC stains were performed on Ventana Discovery Ultra (Ventana Medical Systems, Roche Diagnostics, Indinapolis, USA) IHC Auto stainer. Briefly, the tissue samples were sectioned at 5 μm and put on positively charged glass slides. The slides were loaded on the machine, deparaffinization, rehydration, endogenous peroxydase activity inhibition and antigen retrieval were first performed. For triple-plex IHC, the three antigens were sequentially detected, and heat inactivation was performed to prevent any cross-reactivity between each antigen detection. Following each primary antibody incubation, DISCOVERY anti-Rabbit HQ or NP and DISCOVERY anti-HQ-HRP or anti-NP-AP were incubated. The stains were then visualized by DISCOVERY Yellow Kit, DISCOVERY purple Kit and DISCOVERY Teal Kit (Ventana), respectively; counterstained with haematoxylin (Ventana) and coverslipped. The following is primary antibody and corresponding color information for triple-plex IHC: USP22-Purple: Clone# EPR18945, Rabbit monoclonal antibody from Abcam Ki-67-Teal: Clone# 30 − 9, Rabbit monoclonal antibody from Ventana, Cleaved caspase-3-Yellow: Clone# ASP135, Rabbit monoclonal antibody from Cell signaling. The single IHC stains were visualized by DISCOVERY ChromoMap DAB Kit (Ventana). USP7-DAB (Brown): Rabbit polyclonal antibody from Genetex CD31-DAB (Brown): Clone# EP78, Rabbit monoclonal antibody from Eptomics. IHC stained slides were digitalized and documented by NanoZoomer S360 Digital Slide Scanner (Hamamatsu) and viewed by NDP.view image viewer software.

### In vivo xenograft and P5091 treatment

The study was reviewed and approved by the Institutional Animal Care and Use Committee (IACUC, #16,005) of City of Hope National Medical Center. All animal protocols were performed in the animal facility at City of Hope National Medical Center in accordance with federal, local, and institutional guidelines. NOD/SCID/IL2Rgamma null mice (NSG) mice (Jackson Labs, Bar Harbor, ME; 24–27 g, 6–8 weeks of age) were used for xenograft experiment. A suspension of 5 × 10^6^ A549, USP22-Ko A549 cancer cells in 0.1 ml RPMI 1640 was injected into the subcutaneous dorsa of mice at the proximal midline. When the tumor volume was about 90–110 mm^3^, mice were randomized into two treatment groups, and each group included 5 mice. For the animal study, mice bearing human A549 xenograft were treated with intraperitoneal injection of vehicle or 10 mg/kg P5091 (4% NMP, 3% Tween-80 and 20% PEG400 with Milli-Q water) once a day for 3 consecutive weeks, as described previously [41]. The weight of the mice was recorded, and tumor volumes were measured and calculated [0.5 × (long dimension) × (short dimension)^2^] 2–3 times a week throughout the study. All animals were euthanized before tumors reached 3000 mm^3^ or showed signs of impending ulceration. At the end of the experiment, xenograft tissues were removed and weighted. Sections will be subjected to Haematoxylin and Eosin (H&E), and IHC stains of USP22, USP7, CD31: endothelial marker for angiogenesis, Ki67: cell proliferation marker; and apoptosis marker: cleaved caspase-3 etc. Quantitative analysis and scoring of quantity and intensity in relation to positive and negative control was performed microscopically using FIJI image analysis software [42, 43]. Microvessel density (MVD) in lung cancer tissues was evaluated after immunostaining endothelial cells with antibody against mouse CD31 (The JC70 Mab from DAKO), and MVD count was carried out on three fields (×100) chosen within the whole vascularized areas. Any endothelial cell or cluster of endothelial cells positive for CD31 was counted as described previously [44].

### Statistical analysis

All experiments were performed in duplicates or triplicates and repeated at least two times in each experiment. Two group comparisons were analyzed for variation and significance using a student’s *t-*test or Pearson χ^2^ test. All data presented is mean ± standard deviation (SD). The correlation between two groups was analyzed by Spearman’s rank correlation analysis. Statistical significance was set at *P <* 0.05.

## Results

### Targeting USP7 significantly upregulated USP22 in cancer cells

**Fig. 1:**
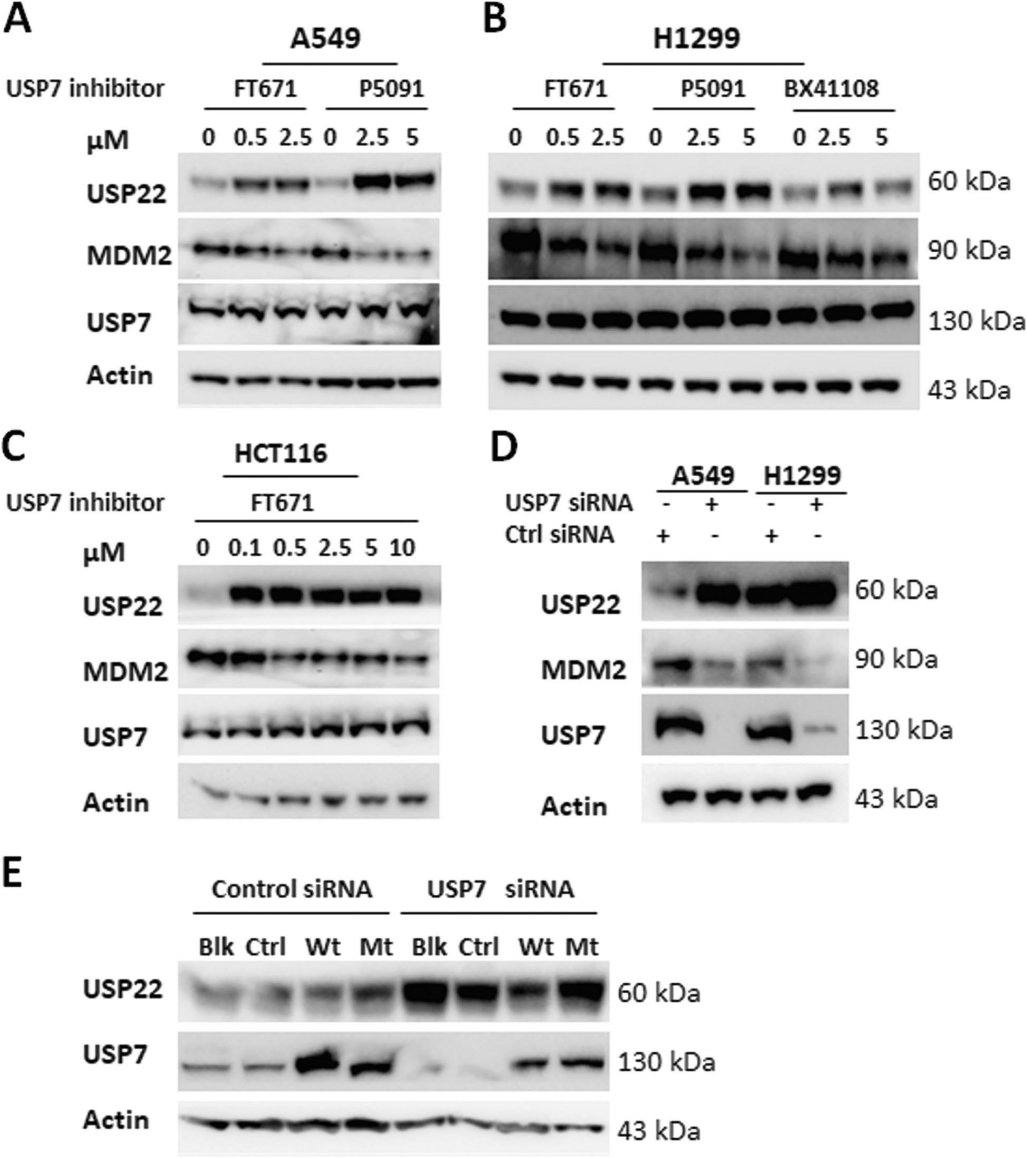
USP7 inhibition upregulated USP22 in cancer cells. USP7 inhibitors FT671, P5091, and HBX41108 treatment upregulated USP22 in **A**. A549. **B**. H1299 and **C**. HCT116 cells in a dose-dependent manner. **D**. Knockdown of USP7 upregulated USP22 in A549 and H1299 lung cancer cells (Ctrl siRNA for control scramble siRNA). Protein was extracted from cells treated with USP7 inhibitors or siRNA for 72h for Western blot analysis. **E**. The upregulation of USP22 is dependent on the inhibition of USP7 deubiquitinase. Western blot analysis of USP22 of H1299 cells in which USP7 was first knocked down by siRNA transfection, 24h later, wild-type USP7 and a catalytically inactive USP7 mutant (C223S) plasmid were individually reintroduced to express the proteins to rescue upregulation of USP22 upon USP7 knockdown (Blk: without plasmid transfection, Ctrl: Control plasmid, Wt: wild type USP7, Mt: C223S loss-of-function USP7 mutant)

We found that the USP7-specific inhibitor FT671 [12] dramatically upregulates USP22 protein in a dose-dependent manner, even at a very low concentration that barely affects cellular proliferation in lung cancer cell line A549 (Fig. 1A). We further tested another two USP7 inhibitors, HBX41108 [45] and P5091 [41], in the lung cancer cell H1299 and colon cancer cell HCT116, and found that both inhibitors dramatically upregulate USP22 in H1299 (Fig. 1B) and HCT116 (Fig. 1C). To further validate the specificity of the upregulation, we made siRNA-mediated USP7-knockdown (USP7-Kd) in A549 and H1299 lung cancer cells and found that USP7-Kd also markedly upregulates USP22 (Fig. 1D) in both cancer cell lines. Consistently, MDM2, a well-known downstream target of USP7 was decreased upon USP7 inhibition or knockdown (Fig. 1A-D). To further confirm the upregulation of USP22 is dependent on the inhibition of USP7 deubiquitinase, we first knocked down USP7 by siRNA transfection, 24 h later, reintroduced either a wild-type USP7 or a catalytically inactive USP7 mutant (C223S) by plasmid transfection, 48 h post transfection, proteins were extracted, Western blot analysis showed the reconstitution of the wild type USP7 with deubiquitinase not the mutant USP7 without catalytical activity significantly decreased the upregulation of USP22 upon USP7 knockdown in H1299 cells (Fig. 1E). Therefore, the above data indicates a universal mechanism is responsible for the upregulation of USP22 upon inhibition or knockdown of USP7 in cancer cells.

### Upregulation of USP22 by USP7 inhibition through SP1 degradation

**Fig. 2:**
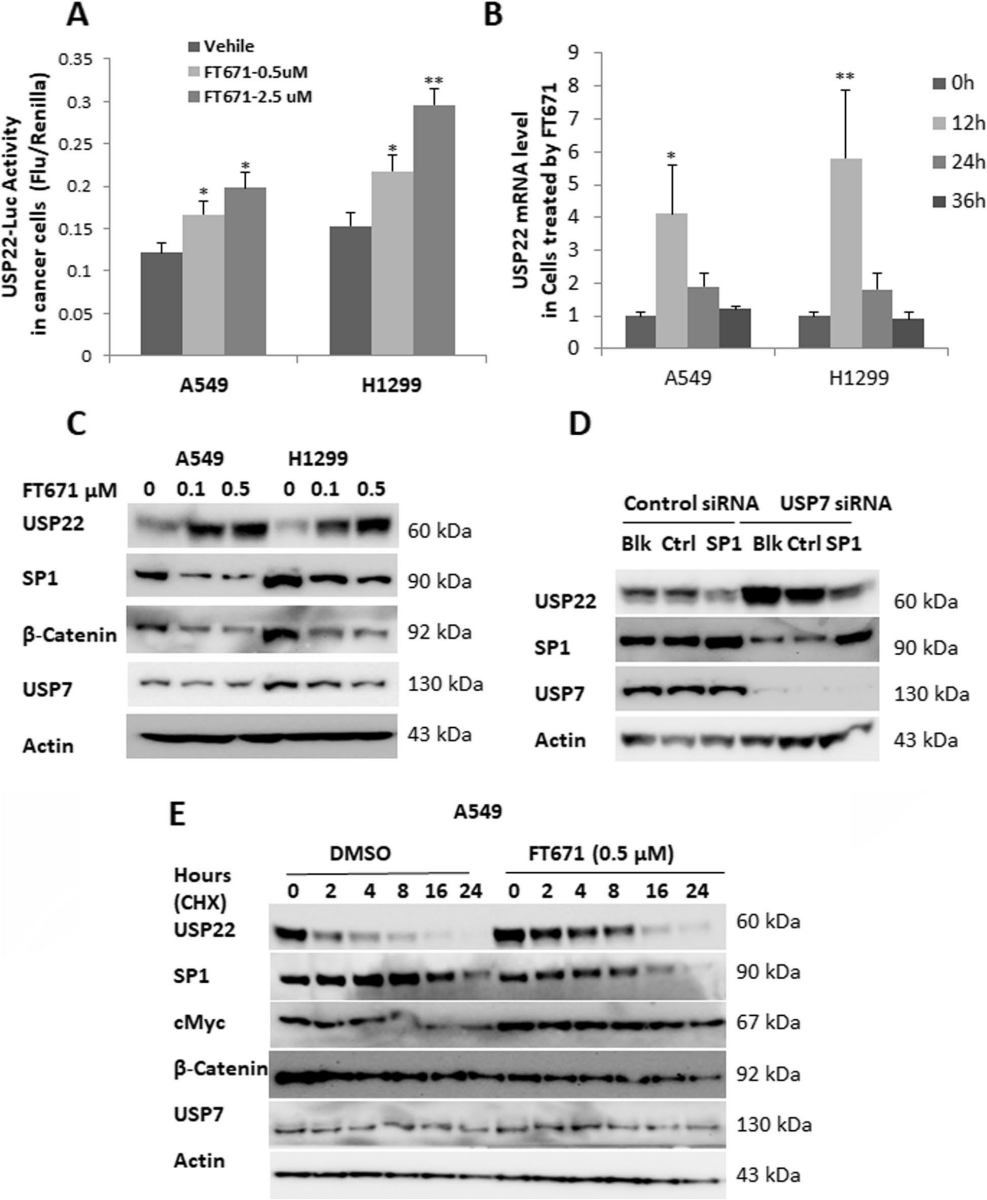
Upregulation of USP22 by USP7 inhibition through SP1 degradation. FT671 treatment significantly elevated **A**. USP22-promoter driven firefly luciferase reporter activity and** B**. Dynamic increase of USP22 mRNA level in both A549 and H1299 lung cancer cells, increase of USP22 mRNA reached maximum at 12h posttreatment, *p<0.05, ** *p*<0.01, compared to Vehicle. **C**. FT671 treatment induced a significant decrease of SP1 and *β*-Catenin in both A549 and H1299 cancer cells. **D**. The upregulation of USP22 by USP7 knockdown is dependent on decrease in SP1 transcriptional activity. Western blot analysis showed that reintroduction of SP1 in USP7-silenced H1299 cells significantly attenuated the upregulation of USP22 by USP7 knockdown. (Blk: without plasmid transfection, Ctrl: Control plasmid, SP1: RSV-SP1 plasmid). **E**. FT671 promoted the degradation of SP1 in A549 cells. A549 cancer cells were pretreated with FT671 for 4 hours, and then treated with 50 µg/ml cycloheximide (CHX) for 0-24h, and the protein was used for Western blot analysis

To explore the underlying mechanism by which USP22 and USP7 expression are inter-related, we first determined the effect of USP7 inhibition on USP22 transcription using a USP22 gene promoter-driven luciferase reporter and RT-PCR analysis, as we recently reported [38]. We found that the addition of 0.5, 2.5 µM of FT671 significantly elevated USP22 luciferase reporter activity (Fig. 2A) and 1.25 µM FT671 induced a dynamic increase of USP22 mRNA level (Fig. 2B) in both A549 and H1299 lung cancer cells. We recently identified that transcription factor SP1 is an inhibitor for USP22 gene expression [38], and here we found that FT671 treatment induced a significant decrease of SP1 protein in both A549 and H1299 cancer cells (Fig. 2C). To demonstrate the upregulation of USP22 by USP7 knockdown is associated with desuppression of SP1 transcriptional activity, USP7 was knocked down, 24 h later, control plasmid and SP1 plasmids were individually introduced to overexpress SP1 in H1299 cells, additional 48 h later, proteins were extracted, Western blot analysis showed that reintroduction of SP1 significantly attenuated the upregulation of USP22 by USP7 knockdown (Fig. 2D). Interestingly, a previous study reported that USP7 regulates the stability of β-Catenin through deubiquitination [46], and we found a dramatic decrease in β-Catenin protein in A549 and H1299 cells treated with USP7 inhibitor FT671 (Fig. 2C). Interestingly, SP1 is complexed with and stabilized by β-Catenin [47], therefore, we hypothesized that FT671-induced SP1 decrease may be via enhanced β-Catenin degradation. Protein stability analysis demonstrated that FT671 significantly promoted the degradation of both SP1 and β-Catenin in A549 cancer cells, indicating this may be one of the mechanisms for decreased SP1 by USP7 inhibition (Fig. 2E). Of note, the stability of c-Myc, a target of USP22, was moderately increased upon USP22 upregulation by USP7 inhibition (Fig. 2E). Taken together, these data suggest that USP7 inhibitor may decrease SP1 via enhancing its degradation with β-Catenin, leading to de-suppressed USP22 transcription and upregulated USP22 expression.

### USP7 inhibitor upregulates c-Myc in cancer cells

**Fig. 3:**
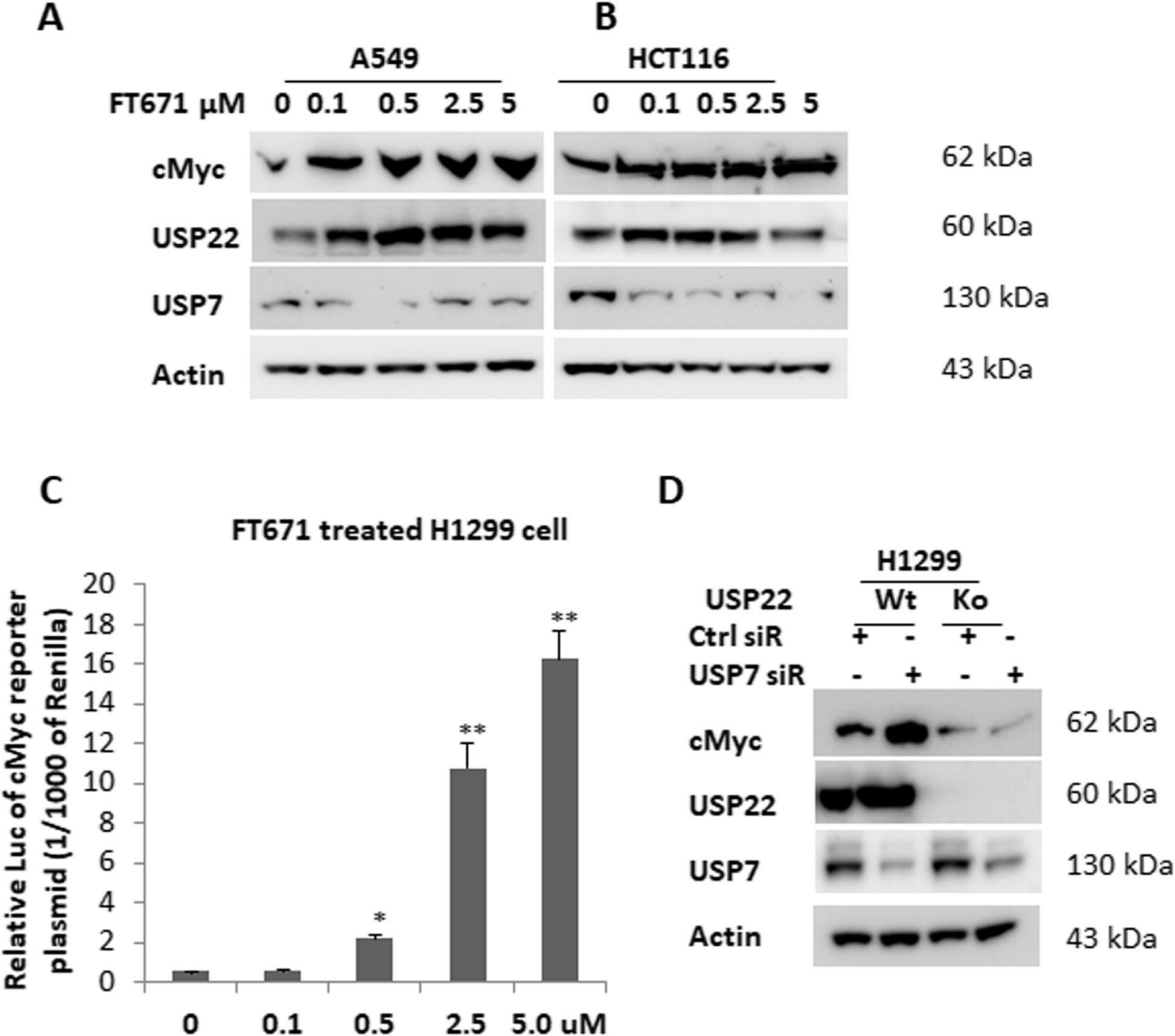
USP7 inhibitor upregulated c-Myc in cancer cells via USP22-dependent way. Western blot analysis of elevated c-Myc in **A**. A549 and **B**. HCT116 cancer cells treated with defined amounts of FT671 for 72h. **C**. FT671 treatment increased c-Myc-responsive luciferase reporter activity in H1299 cells in a dose-dependent manner, *p<0.05, ** *p*<0.01, compared to 0 (Vehicle). **D**. Western blot analysis of FT671-induced c-Myc in the parental (Wt) and USP22-Ko (Ko) H1299 cancer cells

USP22 stabilizes c-Myc and enhances its transcriptional activity through deubiquitination of c-Myc in cancer cells [17, 48]. Given the importance of c-Myc and its contribution to the biological function of USP22, we further examined the change of c-Myc and its activity upon upregulation of USP22 by USP7 inhibition. Western blot analysis showed FT671 significantly upregulated c-Myc  in both lung cancer (Fig. 3A) and in the colorectal cancer cell line HCT116 (Fig. 3B). Simultaneously, elevated USP22 protein was observed in these two cancer cell lines. We used a c-Myc-responsive luciferase reporter assay to study c-Myc pathway activation and found that FT671 significantly increased c-Myc transcriptional activity in H1299 lung cancer cells in a dose-dependent manner (Fig. 3C). Moreover, we validated that c-Myc upregulation is USP22-dependent, as c-Myc upregulation is abolished in USP22-Ko cancer cells (Fig. 3D). Therefore, USP7 inhibition upregulates c-Myc oncogenic activity through USP22, and knockdown of USP22 will counteract this “side effect” of USP7 inhibition.

### Decreased H2Bub1 in USP22+/+ cells & enhanced p53 activation in USP22-Kd cells by USP7 inhibitor

**Fig. 4:**
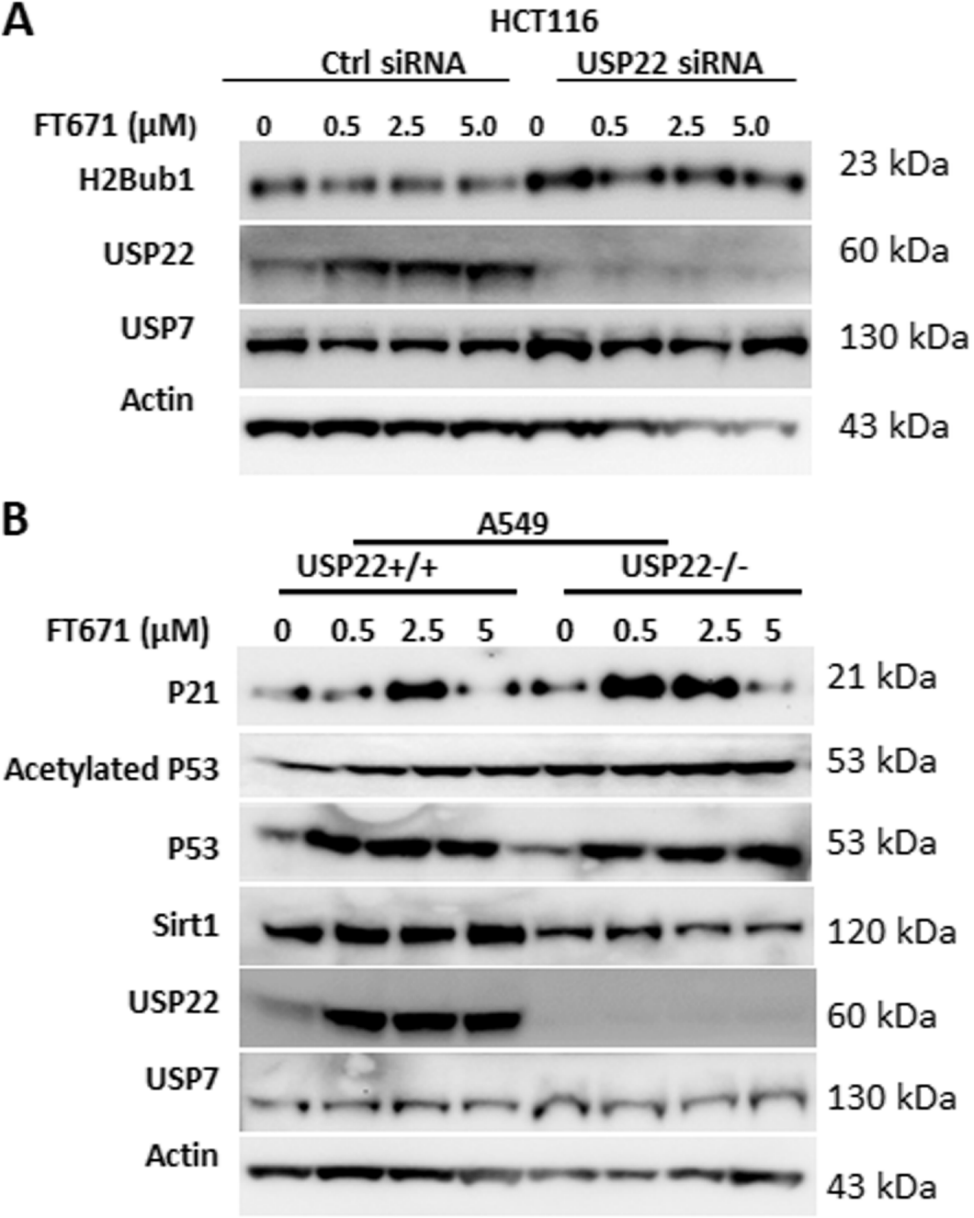
USP7 inhibitor decreased H2Bub1 in USP22+/+ cells and further enhanced p53 activation in USP22-Kd cancer cells. **A**. Impact of FT671 treatment on H2Bub1 in the parental and USP22-Kd HCT116 cancer cells. **B**. Western blot analysis of Sirt1-P53-P21 in the parental (USP22+/+) and USP22-Ko (USP22-/-) A549 cells treated with FT671

H2Bub1 is a well-known key target of USP22 in cancer cells, and it is involved in histone modification and gene expression; loss of H2Bub1 is frequently observed in and associated with more malignant phenotype and poor prognosis of various cancers [49–51]. Here, we found that H2Bub1 level was significantly decreased after treatment with the USP7 inhibitor FT671 in HCT116 cells in a dose-dependent manner (Fig. 4A). However, there was no change in H2Bub1 level in USP22-Kd HCT116 cells in response to FT671 treatment (Fig. 4A), indicating that this change is USP22-dependent. Furthermore, USP7 plays key roles in the p53 tumor suppressor pathway through stabilization of p53 via increasing MDM2, the E3 ligase largely responsible for the ubiquitination of p53 [6, 52]; while USP22 is reported to antagonize p53 transcriptional activation by deubiquitinating Sirt1 which deacytatates p53 and reduces its transcriptional activity [34]. Therefore, we further investigated the effect of USP7 inhibition on p53 pathway activation in parental (USP22-wildtype) and USP22-Ko A549 lung cancer cells with a wild-type p53. Although USP22 is upregulated, and sequentially elevated Sirt1, the change doesn’t dramatically change the increase of the total and acetylated p53, indicating a dominant role of MDM2 in regulation of p53 stability; however, we didn’t observe a dramatic upregulation of P21, a key downstream target of p53 pathway, by the used low dose of FT671 in the parental A549 cells (Fig. 4B). Compared with the parental A549 cells, USP22-Ko A549 has higher levels of the total and acetylated p53 protein; FT671 further increased the acetylated p53. In addition, P21 is significantly upregulated in USP22-Ko cancer by the low dosage of FT671, indicating that a stronger activation of p53 pathway is induced in USP22-Ko cells. These data suggest that targeting both USP7 and USP22 further enhances activation of tumor suppressor p53 pathway in cancer cells, likely resulting in much stronger cell-cycle arrest and apoptosis.

### Targeting USP7 further suppresses cellular growth and sensitizes USP22-Ko lung cancer cells to cisplatin treatment

**Fig. 5:**
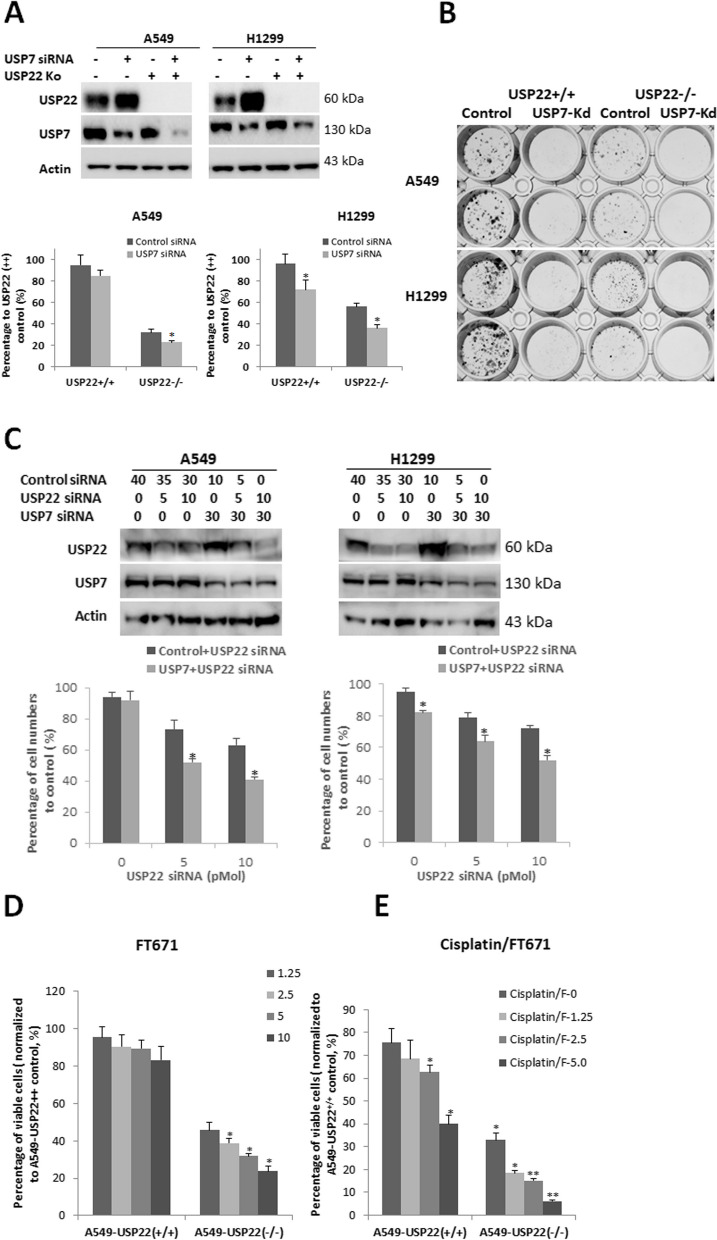
USP7 inhibitor further suppressed cellular growth and sensitized USP22-Ko lung cancer cells to Cisplatin treatment.** A**.Western blot analysis of USP22 and USP7 (Upper panel) and the proliferation (Lower panel) in control siRNA and USP7 siRNA-transfected parental (USP22+/+) or USP22-Ko (USP22-/-) A549 and H1299 cells, *p<0.05, compared to control siRNA. **B**. The colony formation of parental and USP22-Ko A549 and H1299 cells upon USP7 knockdown (USP7-Kd). **C**. Western blot analysis of USP22 and USP7 (Upper panel) and relative cell percentage (Lower panel, normalized to controls transfected with scramble siRNA alone) in cancer cells transfected with a total of 40 pMol of USP22, USP7 or scramble siRNA alone or their combinations at 72h post-transfection, *p<0.05, compared to cell transfected with USP22 siRNA alone. **D**. Treatment of FT671 at 1.25, 2.5, 5, 10 μΜ significantly suppressed the proliferation of USP22-Ko A549 (A549-USP22-/-) cancer cells but not that of the parental A549 cancer cells (A549-USP22+/+). **E**. USP22-Ko A549 cancer cells were more sensitive to FT671 (1.25, 2.5, 5 μM) and its combination with Cisplatin (5 μM), *p<0.05, ** *p*<0.01, compared to 0 (Vehicle) or Cisplatin alone

Based on the above data, we hypothesized that targeting USP7 and USP22 will induce a broad antitumor effect. To test this hypothesis, we first examined if knockdown of USP7 will further suppress USP22-Ko lung cancer cell in vitro proliferation. As shown in Fig. 5A, siRNA-mediated knockdown of USP7 significantly suppressed the parental A549 and H1299 cancer cells, and further suppressed the USP22-Ko A549 and H1299 cells. Consistently, colony formation assay showed the same result (Fig. 5B). Since USP22 is more dominant than USP7 in cancer cell proliferation, USP22 was partially knocked down in A549 and H1299 cancer cells (Fig. 5C, Upper panel), using 1/6 and 1/3 of amounts of siRNA that could completely silence USP22 to further confirm the synergistic effect of targeting these two Dubs; the results showed that partial knockdown of USP22 significantly enhanced the inhibition of USP7 knockdown alone on the proliferation of both cancer cells (Fig. 5C, Lower panel). We next investigated the inhibitory effect of FT671 on in vitro proliferation of the parental and USP22-Ko cancer cells. We found that FT671 only slightly suppressed the parental cancer cell proliferation and significantly suppressed the proliferation of USP22-Ko lung cancer cells (Fig. 5D). We previously demonstrated that USP22 contributes cisplatin chemotherapy resistance, and knockdown of USP22 sensitized lung cancer cells to cisplatin [28, 38], while a previous study found that USP7-silenced A549 cells had enhanced sensitivity to paclitaxel and docetaxel, but there was no significant change in sensitivity toward carboplatin and cisplatin [53]. We hypothesized that USP7-induced USP22 may be responsible for the insensitivity. Notably, we here found that USP22-Ko cells were more sensitive to Cisplatin alone and its combination with USP7 inhibitor, compared to the parental A549 cells, and a more significant proliferation inhibition were induced by the combination in USP22-Ko A549 and H1299 cells (Fig. 5E). Therefore, the above data suggest that targeting both USP7 and USP22 may lead to a synergetic inhibitory effect on cancer cell proliferation and enhanced apoptosis.

### USP7 inhibitor suppresses in vivo growth of USP22-Ko Cells

**Fig. 6:**
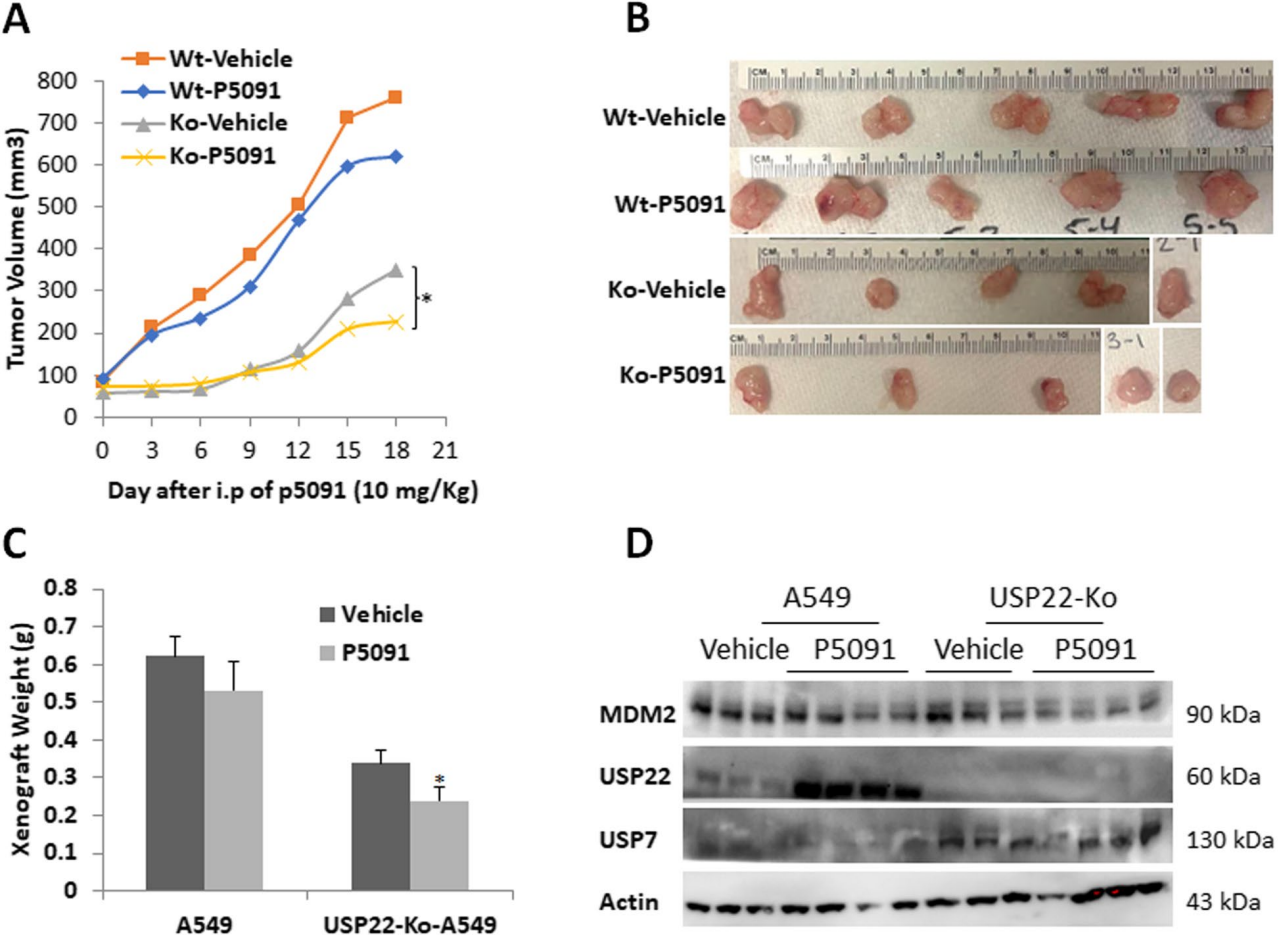
USP7 inhibitor PD5091 suppressed *in vivo* growth of USP22-Ko A549 cancer cells.** A**. In vivo growth curves of these two xenografts treated with vehicle or P5091 (Wt: parental A549; Ko: USP22-Ko-A549). **B**. Cancer xenograft images **C**. Cancer xenograft weights of A549 and USP22-Ko A549 lung cancer cells treated with USP7 inhibitor P5091, *p<0.05, ** *p*<0.01, compared to Vehicle. **D.** Western blot analysis of USP22, MDM2 in A549 and USP22-Ko-A549 lung cancers treated with USP7 inhibitor P5091

We previously showed that USP22-Ko significantly suppressed lung cancer xenograft growth [38]. Based on the above findings, we then hypothesized that targeting USP7 and USP22 may represent a novel effective antitumor therapeutic strategy. To further test this, we generated in vivo xenograft tumors of the parental and USP22-Ko A549 cancer cells in mouse, and then the xenografts were treated with a USP7-specific inhibitor P5091 at the previously reported concentration [16]. Surprisingly, the in vivo experiment data showed that USP7 inhibitor treatment only slightly suppressed the parental A549 cancer xenograft growth, which was not consistent with the in vitro cell culture data. Of note, P5091 treatment did significantly suppress the in vivo growth of USP22-Ko A549 xenografts (Fig. 6A-C). By Western blot analysis, we showed that MDM2 was slightly decreased in both parental and USP22-Ko A549 cancer cells, while USP22 was significantly upregulated by P5091 treatment (Fig. 6D), which confirms that P5091 effectively inhibited USP7 deubiquitinase activity.

### USP7 inhibition induced more apoptosis and suppressed angiogenesis in USP22-Ko cancer cells

**Fig. 7:**
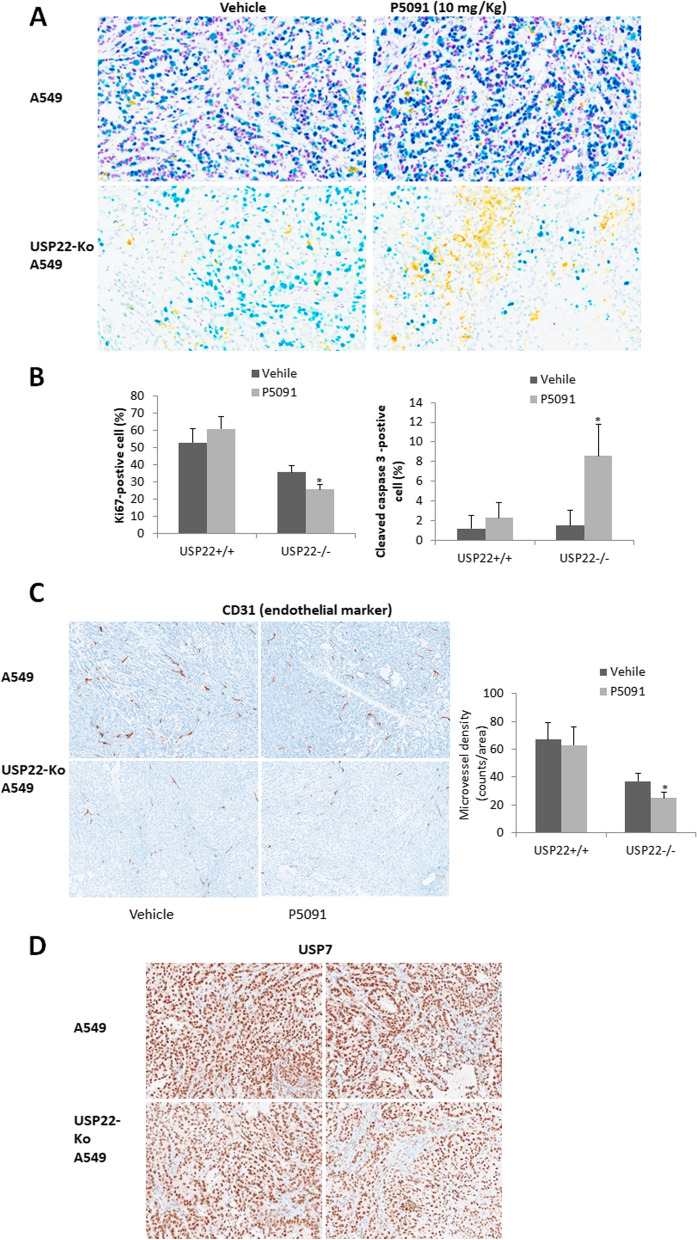
PD5091 induced more apoptosis and suppressed angiogenesis in USP22-Ko cancer cells.** A**. Multiplex IHC analysis of USP22 (Purple), Ki67 (Teal), and cleaved caspase-3 (Yellow) in the parental and USP22-Ko A549 xenograft tissues treated with P5091. **B**. Semi-quantitative analysis of Ki67-positive and cleaved caspase 3- positive cell populations in the parental and USP22-Ko A549 treated with P5091. **C**. IHC analysis of CD31 (Brown) in the parental and USP22-Ko A549 xenograft tissues treated with P5091 (Left panel), and semi-quantitative analysis of MVD in these xenografts (Right panel), *p<0.05, compared to Vehicle. **D**. IHC analysis of USP7 (Brown) in the parental and USP22-Ko A549 xenografts treated with P5091

To further explore how USP7 inhibit USP22-Ko A549 in vivo growth, we did multiple IHC analysis for USP22, Ki67, Caspase 3 in the two xenograft tumors from mice treated with P5091. We found that P5091 treatment barely changed the Ki67 and cleaved caspase-3 in the parental A549 xenografts but did decrease Ki67-positive cells and induced significant more apoptosis in USP22-Ko A549 xenografts (Fig. 7A). Semi-quantitative IHC analysis indicated that Ki67-positive cells were significantly lower (Fig. 7B), while cleaved caspase 3-positively stained area/cells were significantly higher in USP22-KO A549 cancer tissues than the parental A549 cancer tissues (Fig. 7B). We previously demonstrated that USP22-Ko xenografts had significantly less angiogenesis. Here we compared the abundance of angiogenesis in these two xenografts treated with P5091 using CD31 as an endothelium marker. we found that USP7 inhibition didn’t modulate angiogenesis in the parental A549 xenografts but did further decrease angiogenesis in USP22-Ko A549 xenografts (Fig. 7C), while no significant change of USP7 IHC was observed in these xenograft tissues, indicating the effect is mainly through its deubiquitinating activity. Taken together, these data suggest USP7 inhibition can synergize USP22-Ko effect in cancers. This further supports the concept that targeting both USP7 and USP22 may represent a novel and effective anticancer therapeutic strategy.

## Discussion

**Fig. 8:**
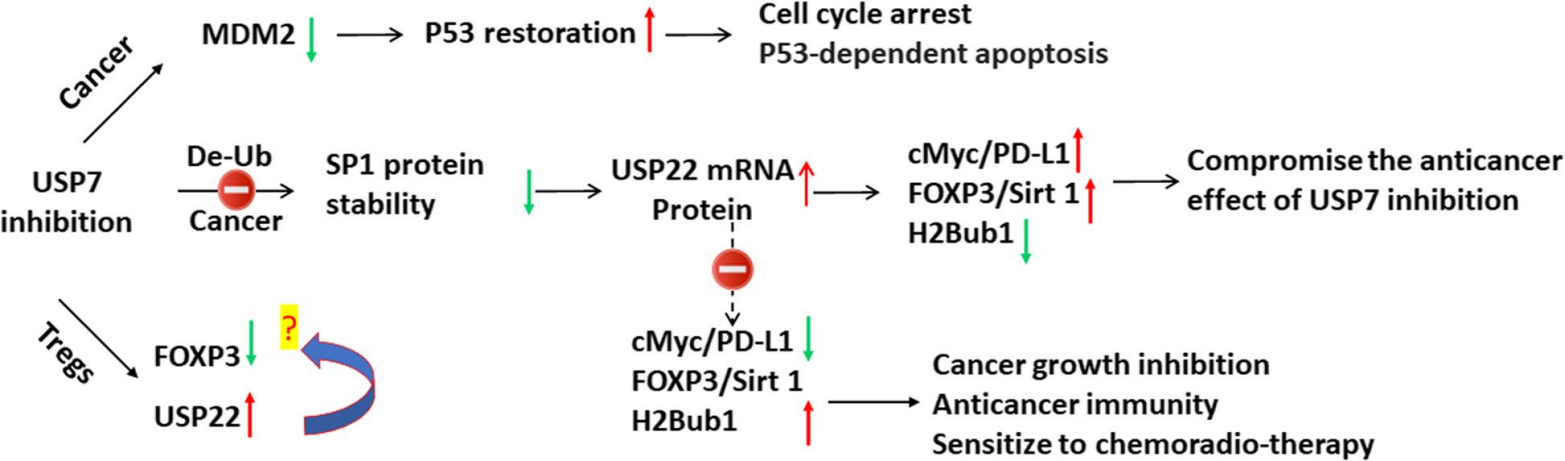
Hypothetic schematic model of response to USP7 inhibition and therapeutic benefits of targeting both USP7 and USP22 in human cancer. Upregulation of USP22 upon USP7 inhibition may activate downstream signaling pathways of USP22 such as c-Myc and H2Bub1 as well as FOXP3 (data not shown) and PD-L1 (data not shown) in cancer cells, which may antagonize antitumor activity of USP7 in human cancer cells, while targeting both USP7 and USP22 will induce broad and synergistic anti-cancer effects

Among Dubs, USP7 is regarded as a highly promising target for variety of cancers due to its critical role in regulating the tumor suppressor p53 signaling pathway along with numerous epigenetic modifiers and transcription factors. Multiple small-molecule inhibitors of USP7 have been reported to exhibit in vitro and in vivo anticancer effect in pre-clinical data [6, 10]. As being summarized in Fig. 8, this study is the first to report that targeting USP7 either by siRNA-mediated knockdown or pharmacological inhibition induces a dramatic transcriptional upregulation of USP22, an important Dub in most cancers and a potential target for cancer treatment. We further demonstrated that targeting both USP7 and USP22 leads to a synergistic effect through modulating multiple important tumor suppressor and oncogenic pathways including p53 and c-Myc, underscoring the therapeutic potential of targeting both USP7 and USP22.

USP22 was early identified as a member of an 11-gene “death-from-cancer” signature that is strongly associated with disease recurrence, metastasis, and treatment failure in a wide range of cancer types, including lung cancer. USP22 catalyzes the removal of the mono-ubiquitin moiety from H2Bub1 and other critical tumor-associated proteins such as c-Myc [17, 48], SIRT1 [34], CCNB1/D1 [54, 55], androgen receptor (AR) [36], BMI1 [36], and FBP1 [56], and has been proven to play multiple and critical roles in cancer growth, stemness, DNA damage response (DDR) [30], resistance to chemotherapy. Recent studies demonstrate that USP22 is a promising target for cancer immunotherapy, since USP22 positively regulates FOXP3 activity in mouse Treg cells that suppress antitumor immune responses, and Treg-specific USP22-knockout (USP22-Ko) suppressed in vivo Treg function to improve antitumor immunity [31]. In addition, USP22 can deubiquitinate and stabilize programmed death-ligand 1 (PD-L1), which mediates immune suppression of cancer cells [32, 33]. Therefore, USP22 is also a promising target for the development of cancer treatments because of its high expression and the critical functions in tumorigenesis of several different carcinomas. A recent study identified a cyclic peptide as the first selective inhibitor of USP22 described [57]; while USP22-specific small-molecule inhibitors aren’t available yet.

The therapeutic potential of USP7 inhibition has been intensively investigated over the last two decades. Preclinical study shows promising results of USP7 inhibitor in treatment of experimental cancer models [15, 16]. Some studies have suggested that the effect of USP7 inhibition may be predominantly through activation of p53. This is evidenced by studies that have shown that USP7 inhibitor treatment accelerated the proteasomal degradation of MDM2 and MDMX in cancer cells, which negatively regulate p53, thereby activating p53 and its downstream target p21, resulting in cell cycle arrest and apoptosis [7, 41, 58]. Recent studies have shown that the effect of USP7 inhibition has important effects on cancer cells that are p53-independent. A recent study demonstrated that USP7 inhibitors generate DNA damage in a p53-independent manner. USP7 inhibition also induces a widespread activation of CDK1 throughout the cell cycle, which leads to DNA damage [59]. Another study suggested that oxidative and endoplasmic reticulum (ER) stress contributes to USP7 inhibitor-mediated apoptosis in cancer cells [14]. A major concern of the application of USP7 inhibitors is their selectivity and their relatively narrow dynamic therapeutic range in anticancer therapy. USP7 inhibitors may target both related and unrelated enzymes such as other homologous DUB [60, 61], which results in unwanted side effects. In the study, we found that USP7 inhibition by three inhibitors: HBX4418 [45], P5091 [41], and FT671 [12] dramatically upregulates USP22 in cancer cells through transcriptional mechanisms, and given the critical role of USP22 in carcinogenesis and anticancer response, we propose that the upregulated USP22 may represent a critical side-effects of USP7 inhibition.

We further examined the expression and activation of downstream signaling of USP22 pathway by USP7 inhibition and found that this feedback upregulation has a significant impact on USP22 pathway and USP22-related oncogenes. First, we found that upregulated USP22 will significantly activate c-Myc oncogenic activity. USP22 can stabilize proto-oncogene c-Myc [17, 48], which plays a critical role in growth control, differentiation, and apoptosis and is frequently overexpressed and activated in human cancer. Given the importance of c-Myc and its contribution to biological function of USP22, we further confirmed that USP7 inhibition upregulates c-Myc oncogenic activity through USP22, and knockdown of USP22 will counteract this “side effect” of USP7 inhibition. Second, we also examined the impact of USP7 inhibition through upregulation of USP22 on H2Bub1, which is a well-known and key target of USP22 in cells and is associated with more malignant phenotype and poor prognosis of various cancers [49–51]. We here found that FT671 induced a significant decrease of H2Bub1 in a dose-dependent manner in colorectal cancer cells. Third, since USP7 has key roles in the p53 tumor suppressor pathway through stabilization of p53 via increasing MDM2 [6, 52], while USP22 is reported to antagonize p53 transcriptional activation by deubiquitinating Sirt1 to suppress [34], we further investigated p53p53 the effect of USP22 and USP7 inhibition on the p53 pathway. We found that USP22-Ko cancer cells have higher activation of p53 and are more sensitive to a combination of cisplatin and USP7 inhibitor. Most importantly, through comparing the in vivo response of the parental and USP22-Ko cancer xenografts to USP7 inhibitor P5091, we found that USP7 inhibitor treatment suppressed more growth and induced more apoptosis in USP22-Ko xenograft, which further support that targeting both USP7 and USP22 may represent a novel and effective anticancer therapeutic strategy. P5091 didn’t significantly suppress A549 lung cancer growth, we conjectured this might be caused by the extremely poor solubility of P5091, and an extended treatment might gain a significant result. Lastly, both USP7 and USP22 are involved in immunosuppression through stabilizing immune check-point inhibitor PD-L1 in cancer and FOXP3 in Tregs, and the significance of this feedback deserves to further study using immune-component cancer model.

In summary, in this study, we have identified a “feedback” of upregulation of USP22 upon USP7 inhibition in cancer cells. Given the essential roles of both USP22 and USP7 in cancer growth, stemness, DDR, and critical cancer-related oncogenic and tumor suppressor signaling axes such as p53, c-Myc, our findings suggest targeting both USP22 and USP7 will induce broad and synergistic anti-cancer activities, which warrant further investigation.

## Data Availability

Not applicable.

## Abbreviations

DUBs: Deubiquitinases
UPS: Ubiquitin–proteasome system
USP7: Ubiquitin-specific protease 7
USP22: Ubiquitin-specific protease 22
USP22-Ko: USP22-knockout
H2Bub1: Mono-ubiquitin moiety from lysine 120 of H2B
CSC: Cancer stem cells
DDR: DNA damage response
IHC: Immunohistochemistry
H&E: Haematoxylin and Eosin
PD-L1: Programmed death-ligand 1
Treg: T regulatory cells
